# Concurrent amplification of Zika, chikungunya, and yellow fever virus in a sylvatic focus of arboviruses in Southeastern Senegal, 2015

**DOI:** 10.1186/s12866-020-01866-9

**Published:** 2020-06-26

**Authors:** Diawo Diallo, Gamou Fall, Cheikh Tidiane Diagne, Alioune Gaye, Yamar Ba, Ibrahima Dia, Ousmane Faye, Mawlouth Diallo

**Affiliations:** 1 Pôle de Zoologie Médicale, Institut Pasteur de Dakar, 36 Avenue Pasteur, BP 220, Dakar, Senegal; 2 Pôle de Virologie, Institut Pasteur de Dakar, 36 Avenue Pasteur, BP 220, Dakar, Senegal

**Keywords:** Zika virus, Chikungunya virus, Yellow fever virus, Amplification, Concurrent, Mosquito vectors, Southeastern Senegal

## Abstract

**Background:**

Chikungunya (CHIKV), yellow fever (YFV) and Zika (ZIKV) viruses circulate in sylvatic transmission cycles in southeastern Senegal, where they share common hosts and vectors. All three viruses undergo periodic *amplifications*, during which they are detected in mosquitoes and sometimes in hosts. However, little is known about their spatio-temporal patterns in years in which they undergo concurrent amplification. The aim of this study was to describe the co-amplification of ZIKV, CHIKV, and YFV, and the daily dynamics of these arboviruses and theirs vectors within villages in southeastern Senegal.

**Results:**

Mosquitoes were collected monthly from July to December 2015. Each evening, from 6 to 9 PM, landing collections were performed by teams of 3 persons working simultaneously in 70 sites situated in forest (canopy and ground), savannah, agriculture, barren, and village (indoor and outdoor) land covers. Collections within villages were continued until 6 AM. Mosquitoes were tested for virus infection by virus isolation and RT-PCR. Seventy-five mosquito pools comprising 10 mosquito species contained at least one virus. *Ae. furcifer* and *Ae. luteocephalus* were infected by all three viruses, *Ae. taylori* by YFV and ZIKV, and remaining seven species by only, only YFV or only ZIKV. No single mosquito pool contained more than one virus. CHIKV was the only virus detected in all land cover classes and was found in the greatest number of sampling sites (32.9%, *n* = 70). The proportion of sites in which more than one virus was detected was less than 6%. *Ae. aegypti formosus*, *Ae. furcifer*, *Ae. luteocephalus*, *Ae. minutus*, *Ae. vittatus*, and *An. gambiae* were found within villages. These vectors were mainly active around dusk but *Ae. furcifer* was collected until dawn. All viruses save ZIKV were detected indoors and outdoors, mainly around dusk. Virus positive pools were detected over 2, 3 and 4 months for YFV, CHIKV and ZIKV, respectively.

**Conclusion:**

Our data indicate that the distribution of different vector species and different arboviruses vary substantially between sites, suggesting that CHIKV, YFV, and ZIKV may have different transmission cycles in Southeastern Senegal.

## Background

Southeastern Senegal is known as an enzootic focus of several arboviruses of medical and veterinary importance [1]. Indeed since 1972 more than 39 viral species have been isolated from mosquitoes, pheblotomes or rodents during arbovirus surveillance programs in the area [2–4], including yellow fever and Zika (YFV and ZIKV, *Flaviviridae*: *Flavivirus*) and chikungunya (CHIKV, genus *Alphavirus,* family *Togaviridae*). These three viruses are mainly transmitted between arboreal *Aedes* vectors and non-human primates in this area, where they utilize an overlapping suite of vectors and hosts. However, some studies suggest the possible involvement of additional vertebrate and mosquito species in secondary transmission cycles of these arboviruses [5]. Human are not indispensables hosts in the sylvatic cycle of these arboviruses but may become infected during activities inside forests or directly within villages, where *Ae. furcifer* and *Ae. vittatus* have been found infected and feeding readily on humans [4, 6, 7].

These arboviruses have recently undergone range expansions, producing frequent and devastating outbreaks in Africa, America, Asia and Europe [8–11]. They are transmitted between human by mainly *Ae. aegypti* and secondarily *Ae. albopictus* [12–14].

Despite the fact that they share the same epidemiological system, these arboviruses showed some differences in their use of mosquito vectors and vertebrate hosts. Additionally, in southeastern Senegal they undergo different amplification cycles, with a 6 year period for YFV and a 4 year period for ZIKV and CHIKV [15], and have generally been amplified in different years. Indeed, high amplification of CHIKV was detected in 1975, 1979, 1983, 1992, 2009; 1977–78, 1983, 1987, 1993, 2001–2 and 2010, YFV amplification was detected in 1973, 1976, 1979–80, 1984–89, 1993, 1997, and ZIKV amplification was detected in 1988, 1990, and 2011 [2, 16, 17]. Although several studies have investigated the amplification of each virus individually, little is known about the dynamics of concurrent amplification of all three viruses (co-amplification). Thus, the aims of this paper are to describe 1) the spatio-temporal dynamics of co-amplification of ZIKV, CHIKV, and YFV in southeastern Senegal in 2015, and 2) the daily dynamics of mosquito vectors and the arboviruses they carry within villages during this co-amplification.

## Results

For the evening sampling in the 70 sites, 10,785 mosquitoes belonging to 7 genera and 48 species were collected and grouped in 1623 pools for viral testing (Table 1). Ten mosquito species were found to be infected by at least one of the three target arboviruses. Among these, *Ae. furcifer* (*n* = 2823), *Ae. dalzieli* (*n* = 2286), *Ae. vittatus* (*n* = 2034), and *Ae. luteocephalus* (*n* = 1107) made 76.5% of the total mosquitoes collected (i.e. both infected and uninfected specimens). The other species found to be positive for at least one virus included *Ae. aegypti formosus* (2.2% of the total mosquito collection), *Ae. minutus* (1.8%), *An. gambiae* (1.7%), *Ma. uniformis* (1.7%), *Ae. taylori* (1.2%), and *Ae. africanus* (1%). Among species comprising more than 1% of the total, only *An. coustani* (3.9%) and *Ae. argenteopunctatus* (2.4%) were not found to carry any of the three target viruses.

**Table 1:** Mosquitoes collected, arboviruses isolated,minimum field infection and entomological inoculation rates of vectors in southeastern Senegal, 2015

Species	Mosquitoes				Viral strains				MFIR‰			BR (F/P/E)	EIR		
	Females	Males	Total	%	CHIKV	YFV	ZIKV	Total	CHIKV	YFV	ZIKV		CHIKV	YFV	ZIKV
*Aedes aegypti formosus*	233	3	236	2.2	2	0	0	2	8.6	0.0	0.0	0.2	0.3	0.0	0.0
*Ae. africanus*	106	0	106	1.0	0	4	0	4	0.0	37.7	0.0	0.1	0.0	0.6	0.0
*Ae. argenteopunctatus*	264	0	264	2.4	0	0	0	0	0.0	0.0	0.0	0.2	0.0	0.0	0.0
*Ae. centropunctatus*	86	0	86	0.8	0	0	0	0	0.0	0.0	0.0	0.1	0.0	0.0	0.0
*Ae. cumminsii*	3	0	3	0.0	0	0	0	0	0.0	0.0	0.0	0.0	0.0	0.0	0.0
*Ae. dalzieli*	2286	0	2286	21.2	0	0	1	1	0.0	0.0	0.4	1.8	0.0	0.0	0.1
*Ae. fowleri*	20	0	20	0.2	0	0	0	0	0.0	0.0	0.0	0.0	0.0	0.0	0.0
*Ae. furcifer*	2763	60	2823	26.2	18	8	9	35	6.5	2.9	3.3	2.2	2.6	1.2	1.3
*Ae. hirsutus*	45	0	45	0.4	0	0	0	0	0.0	0.0	0.0	0.0	0.0	0.0	0.0
*Ae. luteocephalus*	1106	1	1107	10.3	4	7	11	22	3.6	6.3	9.9	0.9	0.6	1.0	1.6
*Ae. mcintoshi*	5	0	5	0.0	0	0	0	0	0.0	0.0	0.0	0.0	0.0	0.0	0.0
*Ae. metallicus*	54	0	54	0.5	0	0	0	0	0.0	0.0	0.0	0.0	0.0	0.0	0.0
*Ae. minutus*	198	0	198	1.8	1	0	0	1	5.1	0.0	0.0	0.2	0.1	0.0	0.0
*Ae. neoafricanus*	2	0	2	0.0	0	0	0	0	0.0	0.0	0.0	0.0	0.0	0.0	0.0
*Ae. ochraceus*	4	0	4	0.0	0	0	0	0	0.0	0.0	0.0	0.0	0.0	0.0	0.0
*Ae. sudanensis*	2	0	2	0.0	0	0	0	0	0.0	0.0	0.0	0.0	0.0	0.0	0.0
*Ae. taylori*	123	7	130	1.2	0	1	1	2	0.0	8.1	8.1	0.1	0.0	0.1	0.1
*Ae. unilineatus*	49	0	49	0.5	0	0	0	0	0.0	0.0	0.0	0.0	0.0	0.0	0.0
*Ae. vittatus*	2006	28	2034	18.9	0	4	0	4	0.0	2.0	0.0	1.6	0.0	0.6	0.0
*Anopheles coustani*	422	0	422	3.9	0	0	0	0	0.0	0.0	0.0	0.3	0.0	0.0	0.0
*An. domicola*	3	0	3	0.0	0	0	0	0	0.0	0.0	0.0	0.0	0.0	0.0	0.0
*An. flavicosta*	36	0	36	0.3	0	0	0	0	0.0	0.0	0.0	0.0	0.0	0.0	0.0
*An. freetownensis*	6	0	6	0.1	0	0	0	0	0.0	0.0	0.0	0.0	0.0	0.0	0.0
*An. funestus*	74	0	74	0.7	0	0	0	0	0.0	0.0	0.0	0.1	0.0	0.0	0.0
*An. gambiae*	183	0	183	1.7	3	0	0	3	16.4	0.0	0.0	0.1	0.4	0.0	0.0
*An. hancocki*	8	0	8	0.1	0	0	0	0	0.0	0.0	0.0	0.0	0.0	0.0	0.0
*An. maculipalpis*	1	0	1	0.0	0	0	0	0	0.0	0.0	0.0	0.0	0.0	0.0	0.0
*An. nili*	9	0	9	0.1	0	0	0	0	0.0	0.0	0.0	0.0	0.0	0.0	0.0
*An. pharoensis*	11	0	11	0.1	0	0	0	0	0.0	0.0	0.0	0.0	0.0	0.0	0.0
*An. rufipes*	19	1	20	0.2	0	0	0	0	0.0	0.0	0.0	0.0	0.0	0.0	0.0
*An. ziemanni*	40	1	41	0.4	0	0	0	0	0.0	0.0	0.0	0.0	0.0	0.0	0.0
*Culex annulioris*	10	0	10	0.1	0	0	0	0	0.0	0.0	0.0	0.0	0.0	0.0	0.0
*Cx. antennatus*	15	4	19	0.2	0	0	0	0	0.0	0.0	0.0	0.0	0.0	0.0	0.0
*Cx. bitaeniorhynchus*	45	0	45	0.4	0	0	0	0	0.0	0.0	0.0	0.0	0.0	0.0	0.0
*Cx. cinereus*	31	9	40	0.4	0	0	0	0	0.0	0.0	0.0	0.0	0.0	0.0	0.0
*Cx. decens*	5	0	5	0.0	0	0	0	0	0.0	0.0	0.0	0.0	0.0	0.0	0.0
*Cx. ethiopicus*	2	0	2	0.0	0	0	0	0	0.0	0.0	0.0	0.0	0.0	0.0	0.0
*Cx. neavei*	13	0	13	0.1	0	0	0	0	0.0	0.0	0.0	0.0	0.0	0.0	0.0
*Cx. nebulosus*	1	0	1	0.0	0	0	0	0	0.0	0.0	0.0	0.0	0.0	0.0	0.0
*Cx. perfuscus*	17	0	17	0.2	0	0	0	0	0.0	0.0	0.0	0.0	0.0	0.0	0.0
*Cx. poicilipes*	30	0	30	0.3	0	0	0	0	0.0	0.0	0.0	0.0	0.0	0.0	0.0
*Cx. quinquefasciatus*	92	6	98	0.9	0	0	0	0	0.0	0.0	0.0	0.1	0.0	0.0	0.0
*Cx. tigripes*	1	0	1	0.0	0	0	0	0	0.0	0.0	0.0	0.0	0.0	0.0	0.0
*Eretmapodites chrysogaster*	3	0	3	0.0	0	0	0	0	0.0	0.0	0.0	0.0	0.0	0.0	0.0
*Er. quinquevittatus*	1	0	1	0.0	0	0	0	0	0.0	0.0	0.0	0.0	0.0	0.0	0.0
*Mansonia africana*	48	0	48	0.4	0	0	0	0	0.0	0.0	0.0	0.0	0.0	0.0	0.0
*Ma. uniformis*	182	0	182	1.7	0	0	1	1	0.0	0.0	5.5	0.1	0.0	0.0	0.1
*Uranotaenia balfouri*	2	0	2	0.0	0	0	0	0	0.0	0.0	0.0	0.0	0.0	0.0	0.0
Total	10,665	120	10,785	100.0											

*%* percentage of the total mosquito collected, *CHIKV* chikungunya virus, *YFV* yellow fever virus, *ZIKV* Zika virus, *MFIR‰* Minimum field infection rate per thousand mosquitoes tested, *BR* Biting rate, *F/P/E* females per person per evening, *EIR* Entomological inoculation rates (number of infected mosquito bites per person between July and December); *Ae*., *Aedes*; *An*., *Anopheles*; *Cx., Culex*; *Er., Eretmapodites*; *Ma*., *Mansonia*

**Fig. 1:**
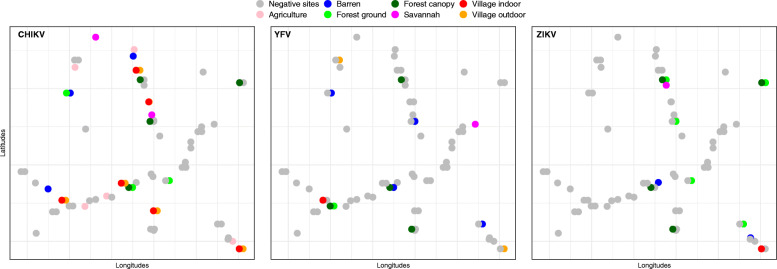
Sampling sites with Zika, chikungunya, and yellow fever viruses positive mosquito pools in southeastern Senegal, 2015

Seventy-five pools were found positive for one of the viruses: ZIKV (23 pools), YFV (20) and or CHIKV (32) (Table 1). Only *Ae. furcifer* (18 pools positive for CHIKV, 8 pools positive for YFV; 9 pools positive for ZIKV) and *Ae. luteocephalus* (4 pools positive for CHIKV, 7 pools positive for YFV, 11 pools positive for ZIKV) were associated with all three viruses. *Ae. taylori* was positive for YFV and ZIKV (1 pool each) while all the other species were positive for only one of the three viruses: CHIKV (*Ae. aegypti formosus* = 2 pools, *Ae. minutus* = 1 pool, and *An. gambiae* = 3 pools), YFV (*Ae. vittatus* and *Ae. africanus*; 4 pools for each species) or ZIKV (*Ae. dalzieli* and *Ma. uniformis*; 1 pool for each species). No mosquito pool was co-infected with more than one virus. The spatio-temporal amplification patterns of viruses varied according to viral species and the vector involved (Fig. 1, Table 2).

**Table 2:** Land cover distribution of arboviruses detected from mosquitoes collected in southeastern Senegal, 2015

Viruses	Land cover class	No. +sites	No. + pools	Species (No. + pools; No. + sites)
CHIKV	Forest canopy	3	7	*Ae. aegypti formosus* (1;1), *Ae. furcifer* (3;1), *Ae. luteocephalus* (3;2)
	Forest ground	3	3	*Ae. furcifer* (2;2), *Ae. luteocephalus* (1;1)
	Agriculture	5	5	*Ae. furcifer* (4;4), *Ae. vittatus* (1;1)
	Barren	2	4	*Ae. furcifer* (2;2), *Ae. vittatus* (2;1)
	Savannah	2	1	*Ae. furcifer* (2;2)
	Village indoor	2	4	*Ae. aegypti formosus* (1;1), *Ae. furcifer* (1;1), *Ae. minutus* (1;1), *Ae. vittatus* (1;1)
	Village outdoor	6	7	*Ae. furcifer* (4;4), *An. gambiae* (3;3)
	Forest canopy	4	7	*Ae. africanus* (2;1), *Ae. furcifer* (1;1), *Ae. luteocephalus* (3;2), *Ae. taylori* (1;1)
	Forest ground	5	9	*Ae. africanus* (2;2), *Ae. furcifer* (3;3), *Ae. luteocephalus* (4;3)
	Agriculture	0	0	
YFV	Barren	0	0	
	Savannah	1	1	*Ae. furcifer* (1;1)
	Village indoor	1	1	*Ae. furcifer* (1;1)
	Village outdoor	2	2	*Ae. furcifer* (2;2)
	Forest canopy	5	9	*Ae. furcifer* (3;3), *Ae. luteocephalus* (5;4), *Ae. dalzieli* (1;1)
	Forest ground	5	8	*Ae. furcifer* (3;3), *Ae. luteocephalus* (3;3), *Ae. taylori* (1;1), *Ma. uniformis* (1;1)
ZIKV	Agriculture	0	0	
	Barren	2	3	*Ae. furcifer* (2;2), *Ae. luteocephalus* (1;1)
	Savannah	1	1	*Ae. luteocephalus* (1;1)
	Village indoor	1	2	*Ae. furcifer* (1;1), *Ae. luteocephalus* (1;1)
	Village outdoor	0	0	

*No. +sites* Number of positives sites, *No. + pools* Number of pools found positive, *CHIKV* chikungunya virus, *YFV* yellow fever virus, *ZIKV* Zika virus; *Ae*., *Aedes*; *An*., *Anopheles*; *Ma*., *Mansonia*

For CHIKV, the mean MFIR per thousand pooled mosquitoes (Table 1) that varied between 3.6 ‰ for *Ae. luteocephalus* and 16.4 ‰ for *An. gambiae* were comparable (Fisher’s Exact Test: *p* **=** 0.2). The infection rates of the vectors varied significantly for YFV (Fisher’s Exact Test: *p* **=** 0.0006) and ZIKV (Fisher’s Exact Test: *p* **=** 0.0003). Differences in MFIR of vectors were due to the higher infection rates of *Ae. africanus* and *Ae. taylori* for YFV and the lower infection rate of *Ae. dalzieli* for ZIKV. The mean entomogical inoculation rates (Table 1), indicate that the main vectors were *Ae. furcifer* (64.5% of the transmission), *Ae. luteocephalus* (14.3%), *An. gambiae* (10.7%) and *Ae. aegypti formosus* (7.1%) for CHIKV, *Ae. furcifer* (33.3%), *Ae. luteocephalus* (29.2%), *Ae. africanus* and *Ae. vittatus* (16.7% each) for YFV, *Ae. luteocephalus* (47.8%) and *Ae. furcifer* (39.1%) for ZIKV.

As shown in Fig. 1 and Table 2, CHIKV was detected in all land cover classes, while YFV was not detected in agriculture or barren land and ZIKV was not detected in agriculture or outdoors in villages. Moreover, CHIKV, which was was found in 32.9% of the 70 sampling sites, was detected significantly more often than the other two viruses (χ^2^ = 18.5, *df* = 2, *p* < 0.001). ZIKV was detected in 20% of sites and YFV in 18.6% of sites, with statistically comparable detection frequencies (χ^2^ = 0, *df* = 1, *p* = 1). While 55.7% of the sampling sites were found positive for at least one virus, the proportion of sites with co-occurrence of 2 or 3 viruses were low and comparable (Fisher’s Exact Test: *p* = 0.8), ranging from about 1 to 5%.

MFIR of each vector species for CHIKV, YFV, and ZIKV (Table 3) were not significantly different between landcover classes investigated (Fisher’s Exact Test: *p* **>** 0.06), except *Ae. luteocephalus* for ZIKV (Fisher’s Exact Test: *p* **=** 0.03). The infection rate of the *Ae. luteocephalus* population collected in villages indoor was significantly higher compared to the other populations. Species involved and their relative importance in the transmission varied by virus and landcover class (Table 3).

**Table 3:** Minimum field infection and entomological inoculation rates of arbovirus vectors in southeastern Senegal, 2015

Virus	Land cover class	Species (MFIR ‰; EIR)
CHIKV	Forest canopy	*Ae. aegypti formosus* (55.6; 1.02), *Ae. furcifer* (4.06; 3.05), *Ae. luteocephalus* (4.92; 3.05)
	Forest ground	*Ae. furcifer* (6.27; 2.03), *Ae. luteocephalus* (2.6; 1.02)
	Agriculture	*Ae. furcifer* (13.38; 4.07), *Ae. vittatus* (1.89; 1.02)
	Barren	*Ae. furcifer* (4.57; 2.04), *Ae. vittatus* (3.16; 2.03)
	Savannah	*Ae. furcifer* (5.76; 2.03)
	Village indoor	*Ae. aegypti formosus* (50; 1.02), *Ae. furcifer* (5.92; 1.02), *Ae. minutus* (166.67; 1.02), *Ae. vittatus* (52.63; 1.02)
	Village outdoor	*Ae. furcifer* (8.83; 4.07), *An. gambiae* (44.11; 3.05)
	Forest canopy	*Ae. africanus* (40; 2.03), *Ae. furcifer* (1.35; 1.01), *Ae. luteocephalus* (4.92; 3.05), *Ae. taylori* (11.9; 1.02)
	Forest ground	*Ae. africanus* (36.36;2.03), *Ae. furcifer* (9.4; 3.05), *Ae. luteocephalus* (10.42; 4.07)
	Agriculture	
YFV	Barren	
	Savannah	*Ae. furcifer* (2.88; 1.02)
	Village indoor	*Ae. furcifer* (5.92; 1.02)
	Village outdoor	*Ae. furcifer* (4.41; 2.03)
	Forest canopy	*Ae. furcifer* (4.06; 3.05), *Ae. luteocephalus* (8.2; 5.09), *Ae. dalzieli* (3.28; 1.02)
	Forest ground	*Ae. furcifer* (9.4; 3.05), *Ae. luteocephalus* (7.81; 3.05), *Ae. taylori* (47.62; 1.02), *Ma. uniformis* (66.67; 1.02)
ZIKV	Agriculture	
	Barren	*Ae. furcifer* (4.57; 2.04), *Ae. luteocephalus* (25.64; 1.02)
	Savannah	*Ae. luteocephalus* (21.74; 1.02)
	Village indoor	*Ae. furcifer* (5.92; 1.02), *Ae. luteocephalus* (250; 1.02)
	Village outdoor	

*MFIR‰* Minimum field infection rate per thousand mosquitoes tested, *EIR* Entomological inoculation rates (number of infected mosquito bites per person between July and December)

Several vectors including *Ae. aegypti formosus*, *Ae. furcifer*, *Ae. luteocephalus*, *Ae. minutus*, *Ae. vittatus*, and *An. gambiae* were found both indoors and outdoors within villages. These vectors were mainly active around dusk between 19 h and 21 h but *Ae. furcifer* was collected in lesser abundance until dawn (Table 4). Both CHIKV and YFV were detected in mosquitoes collected indoors and outdoors, while ZIKV was detected only indoors, mainly around dusk. Only YFV was detected in *Ae. furcifer* collected indoors and outdoors between 21 h and midnight.

**Table 4:** Mosquitoes collected and arboviruses isolated within villages indoors and outdoors in southeastern Senegal, 2015

	Hours	*Ae. aegypti formosus*		*Ae. furcifer*		*An. gambiae*		*Ae. luteocephalus*		*Ae. minutus*		*Ae. vittatus*	
		Outdoor	Indoor	Outdoor	Indoor	Outdoor	Indoor	Outdoor	Indoor	Outdoor	Indoor	Outdoor	Indoor
Number of Mosquito collected	19 h–21 h	96	20	423	199	68	24	5	4	12	6	116	19
	21 h–22 h			33	8								
	22 h–23 h			18	3							1	
	23 h–00 h			17	6								
	00 h–01 h			4	4								
	01 h–02 h			7	4								
	02 h–03 h			8	1				1				
	03 h–04 h			5	2								
	04 h–05 h			3									
	05 h–06 h			1	1								
	Total	96	20	519	228	68	24	5	5	12	6	117	19
Number of viral strains isolated	19 h–21 h		1 CHIKV	4 CHIKV, 2YFV	1 CHIKV,1 YFV,1 ZIKV	3 CHIKV			1 ZIKV		1 CHIKV		1 CHIKV
	21 h–22 h				2 YFV								
	22 h–23 h				1 YFV								
	23 h–00 h			1 YFV									
	00 h–01 h												
	01 h–02 h												
	02 h–03 h												
	03 h–04 h												
	04 h–05 h												
	05 h–06 h												
	Total		1	7	6	3			1		1		1

*CHIKV* chikungunya virus, *YFV* yellow fever virus, *ZIKV* Zika virus; *Ae*., *Aedes*; *An*., *Anopheles*

Seasonally, positive mosquito pools were collected from July to September for CHIKV, October to November for YFV, and August to November for ZIKV (Table 5). The entomogical inoculation rates, indicate that *Ae. vittatus* and *Ae. luteocephalus* were the main vectors of CHIKV in July. This role was played by *Ae. furcifer* the following 2 months. For YFV, *Ae. luteocephalus* and *Ae. furcifer* were the main vectors at the beginning of the transmission in October while *Ae. furcifer* played a main role in November. *Ae. luteocephalus* and *Ae. furcifer* had equal importance in ZIKV transmission between August and October and were supplanted by *Ae. dalzieli* and *Ae. taylori* in November.

**Table 5:** Temporal dynamics of mosquito vectors and arboviruses in southeastern Senegal, 2015

		Species	July	August	September	October	November	December
Total mosquitoes collected		*Ae. aegypti formosus*	132	54	18	13	16	0
		*Ae. africanus*	0	0	0	80	24	2
		*Ae. dalzieli*	18	4	26	1120	1106	12
		*Ae. furcifer*	1436	230	339	533	219	6
		*An. gambiae*	8	63	78	10	23	1
		*Ae. luteocephalus*	608	88	180	179	48	3
		*Ae. minutus*	117	6	75	0	0	0
		*Ae. taylori*	47	6	26	26	17	1
		*Ma. uniformis*	2	1		39	138	2
		*Ae. vittatus*	1438	193	270	84	21	0
		Total	3806	645	1012	2084	1612	27
Landing Rates		*Ae. aegypti formosus*	0.63	0.26	0.09	0.06	0.08	0.00
		*Ae. africanus*	0.00	0.00	0.00	0.38	0.11	0.01
		*Ae. dalzieli*	0.09	0.02	0.12	5.33	5.27	0.06
		*Ae. furcifer*	6.84	1.10	1.61	2.54	1.04	0.03
		*An. gambiae*	0.04	0.30	0.37	0.05	0.11	0.00
		*Ae. luteocephalus*	2.90	0.42	0.86	0.85	0.23	0.01
		*Ae. minutus*	0.56	0.03	0.36	0.00	0.00	0.00
		*Ae. taylori*	0.22	0.03	0.12	0.12	0.08	0.00
		*Ma. uniformis*	0.01	0.00	0.00	0.19	0.66	0.01
		*Ae. vittatus*	6.85	0.92	1.29	0.40	0.10	0.00
Number of viral strains	CHIKV	*Ae. aegypti formosus*	1	1	0	0	0	0
		*Ae. furcifer*	1	6	11	0	0	0
		*An. gambiae*	0	1	2	0	0	0
		*Ae. luteocephalus*	2	1	1	0	0	0
		*Ae. minutus*	1	0	0	0	0	0
		*Ae. vittatus*	3	1	0	0	0	0
	YFV	*Ae. africanus*	0	0	0	2	2	0
		*Ae. furcifer*	0	0	0	4	4	0
		*Ae. luteocephalus*	0	0	0	6	1	0
		*Ae. taylori*	0	0	0	0	1	0
	ZIKV	*Ae. dalzieli*	0	0	0	0	1	0
		*Ae. furcifer*	0	2	5	2	0	0
		*Ae. luteocephalus*	0	2	7	2	0	0
		*Ae. taylori*	0	0	0	0	1	0
		*Ma. uniformis*	0	0	0	1	0	0
MFIR‰	CHIKV	*Ae. aegypti formosus*	0.01	0.02	0.00	0.00	0.00	0.00
		*Ae. furcifer*	0.00	0.03	0.03	0.00	0.00	0.00
		*An. gambiae*	0.00	0.02	0.03	0.00	0.00	0.00
		*Ae. luteocephalus*	0.00	0.01	0.01	0.00	0.00	0.00
		*Ae. minutus*	0.01	0.00	0.00	0.00	0.00	0.00
		*Ae. vittatus*	0.00	0.01	0.00	0.00	0.00	0.00
	YFV	*Ae. africanus*	0.00	0.00	0.00	0.03	0.08	0.00
		*Ae. furcifer*	0.00	0.00	0.00	0.01	0.02	0.00
		*Ae. luteocephalus*	0.00	0.00	0.00	0.03	0.02	0.00
		*Ae. taylori*	0.00	0.00	0.00	0.00	0.06	0.00
	ZIKV	*Ae. dalzieli*	0.00	0.00	0.00	0.00	0.00	0.00
		*Ae. furcifer*	0.00	0.01	0.01	0.00	0.00	0.00
		*Ae. luteocephalus*	0.00	0.02	0.04	0.01	0.00	0.00
		*Ae. taylori*	0.00	0.00	0.00	0.00	0.06	0.00
		*Ma. uniformis*	0.00	0.00	0.00	0.03	0.00	0.00
EIR	CHIKV	*Ae. aegypti formosus*	0.15	0.15	0.00	0.00	0.00	0.00
		*Ae. furcifer*	0.15	0.89	1.57	0.00	0.00	0.00
		*An. gambiae*	0.00	0.15	0.29	0.00	0.00	0.00
		*Ae. luteocephalus*	0.30	0.15	0.14	0.00	0.00	0.00
		*Ae. minutus*	0.15	0.00	0.00	0.00	0.00	0.00
		*Ae. vittatus*	0.44	0.15	0.00	0.00	0.00	0.00
		All vectors	1.18	1.48	2.00	0.00	0.00	0.00
	YFV	*Ae. africanus*	0.00	0.00	0.00	0.30	0.29	0.00
		*Ae. furcifer*	0.00	0.00	0.00	0.59	0.57	0.00
		*Ae. luteocephalus*	0.00	0.00	0.00	0.89	0.14	0.00
		*Ae. taylori*	0.00	0.00	0.00	0.00	0.14	0.00
		All vectors	0.00	0.00	0.00	1.48	0.86	0.00
	ZIKV	*Ae. dalzieli*	0.00	0.00	0.00	0.00	0.14	0.00
		*Ae. furcifer*	0.00	0.30	0.71	0.30	0.00	0.00
		*Ae. luteocephalus*	0.00	0.30	1.00	0.30	0.00	0.00
		*Ae. taylori*	0.00	0.00	0.00	0.00	0.14	0.00
		*Ma. uniformis*	0.00	0.00	0.00	0.15	0.00	0.00
		All vectors	0.00	0.59	1.71	0.74	0.29	0.00

*MFIR‰* Minimum field infection rate per thousand mosquitoes tested, *EIR* Entomological inoculation rates (number of infected mosquito bites per person per month), *CHIKV* chikungunya virus, *YFV* yellow fever virus, *ZIKV* Zika virus; *Ae*., *Aedes*; *An*., *Anopheles*; *Ma*., *Mansonia*

## Discussion

In this study we describe a co-amplification of ZIKV, YFV, and CHIKV in southeastern Senegal of unprecedented scope. Amplifications of CHIKV [7], YFV [4] and ZIKV [17] were most recently detected in this system in 2009, 2010 and 2011, respectively. These three viruses were last isolated concurrently in 1990 during an outbreak of dengue virus is southeastern Senegal [18]. The low number of strains of these 3 viruses detected in 1990 (3 ZIKV strains, 2 YFV strains and 1 CHIKV strain) may be due to a low-level of circulation of these arboviruses in the sylvatic area or the low number of sites (6 sampling sites) and land cover classes investigated. Interestingly, in the same year (2015) that it was detected in our surveillance system, ZIKV was responsible for huge outbreaks in Brazil [19] and several countries of Latin America [20], and Cabo Verde [21] with the first association of this virus with severe maternal, neonatal and child health complications.

The lack of co-infection of individual mosquito pools with multiple arboviruses could be linked to the low percentage of sites where co-amplification occurred. Experimental work has shown that a single mosquito specimen can be coinfected and effectively transmit two or three arboviruses [22–25]. The low level of co-amplification strongly suggests that these arboviruses have different spatial amplification patterns and probably also different amplification cycles despite the fact that they share the same main vectors (*Ae. furcifer* and *Ae. luteocephalus*) in this and previous studies [4, 6, 7]. The other facts suggesting different amplification cycles of CHIKV, YFV, and ZIKV, includes differences in the role of the main vectors in the landcover classes investigated, number of species involved as vectors (6 CHIKV, 4 YFV, 5 ZIKV), and temporal dynamics of the virus (3 months for CHIKV, 2 for YFV and 4 for ZIKV). The same pattern (with more vectors involved and more months when the infection were detected) was observed in previous studies in the same area when these viruses were detected in 3 different seasons [4, 6, 7]. While not investigated in this study, non-human primates (NHP) are considered as the main vertebrate hosts involved in the sylvatic cycle of CHIKV, YFV, and ZIKV. These NHPs, including *Chlorocebus sabaeus*, *Erythrocebus patas* and *Papio papio*, were found infected by virus isolation and serology in the study area [12, 17]. The role played by these NHPs on the maintenance of these viruses over time was questioned. Thus, investigations on the potential role of other vertebrate hosts detected Avian blood meals from *Ae. furcifer* and *Ae. taylori* [5]. The results also suggested a possible involvement of several wild vertebrates, including birds, rodents, and bats in other transmission cycles of these viruses [17, 26–28]. Thus, these differences in amplification patterns may reflect differences in the community of amplification and reservoir hosts utilized by these viruses.

The host-seeking activities of vectors and transmission of CHIKV, YFV and ZIKV within villages including inside homes, suggest that risk of transmission of sylvatic arboviruses to humans is higher than expected in this population. The involvement of six vector species (*Ae. aegypti formosus*, *Ae. furcifer*, *Ae. minutus*, *Ae. luteocephalus*, *Ae. vittatus* and *An. gambiae*) in arbovirus transmission within villages, including four species (*Ae. aegypti formosus*, *Ae. minutus*, *Ae. luteocephalus*, and *An. gambiae*) detected for the first time in this study, also highlights the high and growing risk of human exposure to arbovirus in domestic environment in southeastern Senegal. It also suggests that all mosquito species present in this domestic environment should be considered potential arbovirus vectors, and their vector competence should be evaluated. The continuous activities of these vectors from dusk to dawn and the transmission of YFV until midnight, after people went to bed, suggest that malaria control interventions including indoor spatial dispersion of insecticides and insecticide-treated nets may be helpful in controlling YFV transmission.

*Aedes furcifer* and *Ae. luteocephalus* pools were tested positive for CHIKV, YFV and ZIKV suggesting a broad susceptibility of these species to arboviruses. Indeed, because they were the most frequently associated to these viruses and among the most abundant species in the sylvatic environment, these species were previously incriminated as the main vectors during sylvatic outbreaks of CHIKV, YFV and ZIKV in southeastern Senegal [2, 4, 6] and several west African countries [17, 29]. However, despite the fact that these two vectors breed mainly in tree-holes [30], the spatial distribution of these vectors suggests that *Ae. furcifer* (found infected in all land cover classes investigated) play an important role both as an epizootic vector (among monkeys in the forest canopies) and a bridge vector (transmitting the viruses to human within villages), whereas *Ae. luteocephalus* played an important role probably only as an epizootic vector [6, 7].

As in the consecutive amplifications of CHIKV, YFV and ZIKV between 2009 and 2011 [4, 6, 7], ZIKV had the longest temporal period of transmission, followed by CHIKV and YFV. For each of these viruses, the duration of transmission was a month shorter compared to the amplifications observed between 2009 and 2011. It is also important to indicate that fewer mosquito species and fewer sampling sites were found infected during this co-amplification compared to the amplifications between 2009 and 2011, with the same sites sampled, suggesting that this co-amplification had a negative impact on the normal dynamics of each of the virus individually.

Our data indicate that mosquito vector activity and arbovirus transmission are highly variable between sites, even those in the same land cover. Mosquito population distribution has been shown to be affected by abundance of their vertebrate blood meal sources [31], thus we speculate that the variation in abundance of particular species may reflect variation in abundance of key host species.

## Conclusion

Our study described the co-amplification of three major arboviruses (ZIKV, CHIKV, and YFV), and the daily dynamics of these arboviruses and theirs vectors within villages in southeastern Senegal. Our results highlighted important spatio-temporal variations in the distribution of the different vectors and arboviruses investigated. Important differences were also observed in the role played by the different vectors in the amplification of the different viruses. These data improve our understanding of the natural histories of arboviruses in the sylvatic environment, and suggest that CHIKV, YFV, and ZIKV may have different transmission cycles in Southeastern Senegal.

## Methods

### Study area

**Fig. 2:**
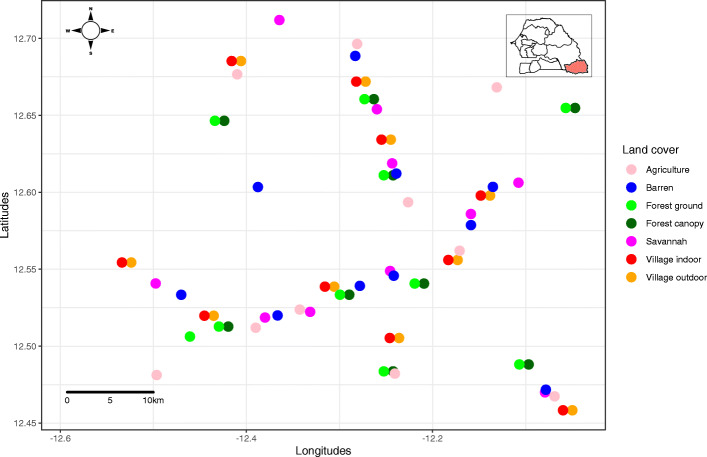
Study sites in southeastern Senegal

Our investigation was done in an area of 1650 km^2^ (30 km in N-S direction; 55 km in E-W direction) around Kedougou city (12°33 N, 12°11 W) in southeastern Senegal (Fig. 2). This study area was extensively described in previous studies [6, 7, 32]. It is located in the fringes of the Fouta Djallon hills, in the borders of Guinea and Mali. The annual rainfall is about 1200 mm and the average temperature is 28 °C. Around 84% of the approximately 156,352 inhabitants of the Kedougou region live in rural areas in small, dispersed villages, and are mainly engaged in agriculture, cattle farming, hunting, and gold mining. Kedougou city is the only large town in the area. The main land cover classes found in the area include forests, savannas, waters, barren lands, agriculture, and villages. The forest land cover is increasingly threatened by human activities including agriculture, charcoal production, gold mining and housing.

### Mosquito sampling

Mosquitoes were collected from July to December 2015 in 70 sites located within the study area. Sites were located in the five main land cover classes of the area (forest, barren, savannah, agriculture, and urban), which were previously classified by remote sensing and geospatial analyses [1, 7, 32–34]. Mosquitoes were collected by human landing collection for one evening (from 6 to 9 PM) per month in each site. Three collectors were used per site in savannah (S), agriculture (A), barren land (B), forest at the ground level (FG), forest in the canopy (FC), villages indoors (VI) and villages outdoors (VO). To better understand the dynamics of vectors and viruses in the domestic environment, mosquito collections in villages were continued until 6 AM. Collection within villages were separated hourly into separate containers.

Mosquitoes were morphologically identified to the species level following appropriate keys [35–40]. Following morphological identification on a chill table, mosquitoes were pooled (maximum of 35 and average 3.9 mosquitoes per pool) by species (one species per pool), sex, and site (one site per pool), and stored in liquid nitrogen in the field and at − 80 °C in the lab for virus testing. No specific permit was needed for sample collection.

### Detection of virus in mosquito pools

After homogenization in 2.5 ml of Leibovit’s L-15 cell culture medium (Gibco BRL, GrandIsland, NY) containing 20% fetal bovine serum (FBS), mosquito pools were centrifuged for 20 min at 10,000 x g at 4 °C for clarification. Viral suspensions were then filtered using a 1 ml syringe (Artsana, Como, Italy), sterilized with 0.20 μm filters (Sartorius, Göttingen, Germany). Viruses isolation attempts were made by inoculating 200 μl of the supernatant of the mosquito homogenate into C6/36 cells (*Ae. albopictus*) as described previously [41]. Viruses were identified, after 7–8 days of incubation, by indirect immunofluorescence with 7 in-house hyper-immune mouse ascites fluids specific to individual or groups of African Flaviviruses, Bunyaviruses, Orbiviruses and Alphaviruses. The identification of viruses were confirmed by complement fixation and seroneutralization tests [4, 6, 7]. All viral tests were carried out by the WHO Collaborating Center of Reference and Research on Arboviruses and hemorrhagic fever viruses of the Institut Pasteur de Dakar.

For the RT-PCR assay, the RNA was extracted from 100 μl of mosquito supernatants using the QiaAmp Viral RNA Extraction Kit (Qiagen, Heiden, Germany) according to the manufacturer’s instructions. RNA was amplified using real-time RT-PCR assay and an ABI Prism 7000 SDS Real-Time apparatus (Applied Biosystems, Foster City, CA) using the Quantitect kit (Qiagen, Hilden, Germany). The real-time RT-PCR protocol (including program, volumes, thermal profiles, primers and sequences probes) were previously described in detail (See Diallo et al. [7] for CHIKV, Diallo et al. [4] for YFV, and Faye et al. [42] for ZIKV).

### Data analysis

For analysis of the distribution of vector species among land cover classes, the mean number of mosquito females collected per site per evening was used as a measure of absolute abundance. The minimum field infection rate (MFIR‰) was calculated as the number of positive per 1000 mosquitoes tested. The entomologic inoculation rate (EIR) defined as the number of infected mosquito bites per human per month, or transmission season (September to December) was also calculated. Differences of frequencies between groups were tested by the χ^2^ or Fisher exact test. All analyses were carried out using R [43].

## Data Availability

All data generated or analysed during this study are included in this published article. Additional data may be available from the corresponding author upon reasonable request.

## Abbreviations

ZIKV: Zika virus
YFV: Yellow fever virus
CHIKV: Chikungunya virus
C6/36: *Aedes albopictus*
S: Savannahs
A: Agricultures
B: Barren lands
FG: Forests at the ground level
FC: Forests in the canopy
VI: Villages indoor
VO: Villages outdoor
MFIR‰: Minimum field Infection Rate
EIR: Entomological Inoculation rate
K-W: Kruskal-Wallis
*Ae.*: *Aedes*
*Cx.*: *Culex*
*An.*: *Anopheles*
*Ma.*: *Mansonia*
*Er.*: *Eretmapodites*
*Ur.*: *Uranotaenia*
